# Management of Chronic Non-healing Wounds by Hirudotherapy

**Published:** 2017-01

**Authors:** Arsheed Iqbal, Afroza Jan, MA Wajid, Sheikh Tariq

**Affiliations:** 1 RRIUM, Naseem Bagh Faculty of Medicine, Kashmir University, Srinagar City, Jammu and Kashmir, India;; 2 JLNM Hospital, Department of plastic surgery, Kashmir University, Srinagar City, Jammu and Kashmir, India

**Keywords:** Hirudotherapy, Wound, Chronic, Healing

## Abstract

A chronic wound is a wound that does not heal in an orderly set of stages and in a predictable amount of time or wounds that do not heal within three months are often considered chronic. Chronic wounds often remain in the inflammatory stage for too long and may never heal or may take years. Chronic wound patients often report pain as dominant in their lives. Persistent pain is the main problem for patients with chronic ulcers. Many wounds pose no challenge to the body’s innate ability to heal; some wounds, however, may not heal easily either because of the severity of the wounds themselves or because of the poor state of health of the individual. Any wound that does not heal within a few weeks should be examined by a healthcare professional because it might be infected, might reflect an underlying disease.

## INTRODUCTION

A chronic wound is a wound that does not heal in an orderly set of stages and in a predictable amount of time or wounds that do not heal within three months are often considered chronic.^1^ Chronic wounds often remain in the inflammatory stage for too long^2^^,^^3^ and may never heal or may take years. Chronic wound patients often report pain as dominant in their lives.^4^^-^^7^ Persistent pain is the main problem for patients with chronic ulcers.^8^ Many wounds pose no challenge to the body’s innate ability to heal; some wounds, however, may not heal easily either because of the severity of the wounds themselves or because of the poor state of health of the individual. Any wound that does not heal within a few weeks should be examined by a healthcare professional because it might be infected, might reflect an underlying disease. 

Wound is a sore on the skin or a mucous membrane, accompanied by the disintegration of tissue. Wound can result in complete loss of the epidermis and often portions of the dermis and even subcutaneous fat. A wound that appears on the skin is often visible as an inflamed tissue with an area of reddened skin. Wound can also be caused due to a lack of mobility, which causes prolonged pressure on the tissues. This stress in the blood circulation is transformed to a skin wound, commonly known as bedsores or decubitus ulcers.^9^

**Fig. 1:**
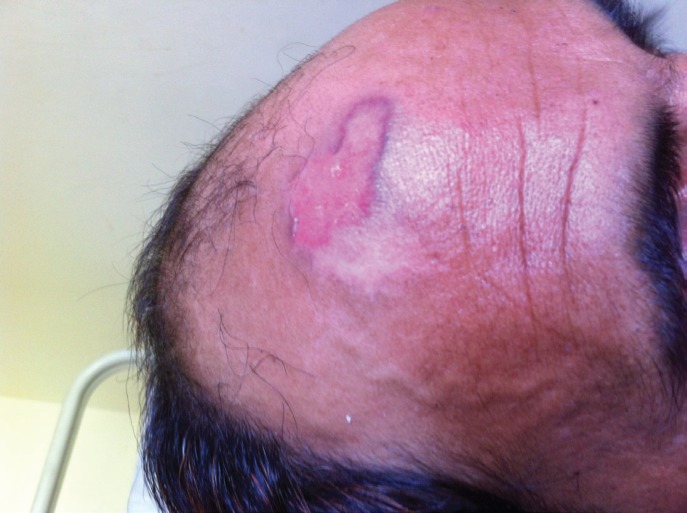
Chronic non-healing ulcer on forehead

Patients may feel pain on the skin around the wound, and fluid may ooze from the wound. In some cases wound can bleed and, rarely patients experience fever. Ulcers develop in stages. In stage 1 the skin is red with soft underlying tissue.^10^ Chronic ulcer symptoms usually include increasing pain, friable granulation tissue, foul odour, and wound breakdown instead of healing (Figure 1).^10^ Ulcers may also appear on the cheeks, soft palate, the tongue, and on the inside of the lower lip. These ulcers usually last from 7 to 14 days and can be painful.^11^

*Etiology*

In addition to poor circulation, neuropathy, and difficulty in moving, there are factors that contribute to chronic wounds include systemic illnesses, age, and repeated trauma. Comorbid ailments that may contribute to the formation of chronic wounds include vasculitis , immune suppression, pyoderma gangrenosum, and diseases that cause ischemia.^2^ Immune suppression can be caused by illnesses or medical drugs used over a long period, for example steroids.^2^ Emotional stress can also negatively affect the healing of a wound, possibly by raising blood pressure and levels of cortisol, which lowers immunity.^5^ Another factor that may contribute to chronic wounds is old age.^6^

Comorbid factors that can lead to ischemia are especially likely to contribute to chronic wounds. Such factors include chronic fibrosis, edema, sickle cell disease, and peripheral artery disease such as by atherosclerosis.^12^ Repeated physical trauma plays a role in chronic wound formation by continually initiating the inflammatory cascade. The wounds from which ulcers arise can be caused by a wide variety of factors, but the main cause is impaired blood circulation. Especially, chronic wounds and ulcers are caused by poor circulation, either through cardiovascular issues or external pressure from a bed or a wheelchair.^13^ Other causes producing skin ulcers include bacterial or viral infections, fungal infections and cancers. Blood disorders and chronic wounds can result in skin ulcers as well.^14^

*Pathophysiology*

Chronic wounds may affect only the epidermis and dermis, or they may affect tissues all the way to the fascia.^7^

*Classification of wounds: Wounds are classified by ‘stage’*

Stage 1 wounds are characterized by redness or discoloration, warmth, and swelling or hardness. Stage 2 wounds partially penetrate the skin. Stage 3 describes full-thickness wounds that do not penetrate the tough white membrane (fascia) separating the skin and fat from the deeper tissues. Stage 4 wounds involve damage to muscle or bone and undermining of adjacent tissue. They may also involve the sinus tracts.^7^

*The stages of wound healing*

All wounds heal in three stages: (i) Inflammatory Stage, occurring during the first few days. The wounded area attempts to restore its normal state by constricting blood vessels to control bleeding. Platelets and thromboplastin make a clot. Inflammation (redness, heat, swelling) also occurs and is a visible indicator of the immune response. White blood cells clean the wound of debris and bacteria. (ii) Proliferative Stage, lasting about 3 weeks (or longer, depending on the severity of the wound). Granulation occurs, which means that special cells called fibroblasts make collagen to fill in the wound. New blood vessels form. The wound gradually contracts and is covered by a layer of skin, and (iii) Maturation and Remodeling Stage, lasting up to 2 years. New collagen forms, changing the shape of the wound and increasing strength of tissue in the area. Scar tissue, however, is only about 80% as strong as the original tissue.

*Wound Infection*

Infection of a wound with a large number of bacteria, a process known as colonization, will slow the healing process. The difference between contamination and colonization is the concentration of bacteria. Anaerobic bacteria such as *Bacteroides*, *Clostridium* and *Streptococcus* may be active at deeper levels of the dermis, insulated from the healing influence of oxygen. Anaerobic bacteria are responsible for many devastating infections resulting in gangrene. Aerobic bacteria are more closely identified with superficial epidermal layers but may also be involved in infective processes and include *Staphylococcus epidermis*, *Corynebacteria*, and *Propionibacteria*.^14^

*Excessive use of antibiotics in non-healing ulcers*

Although approximately 4 million cases of non-healing ulcers are diagnosed annually in Europe,

non-healing ulcers have been considered a negligible problem in society.^14^ patients with non-healing ulcers is an excess usage of antibiotics. As early as 1998, it was reported that 60.1% of all ulcer patients were treated with at least one antibiotic within a six-month period.^15^

*Microbiology of chronic ulcers*

Conditions such as chronic venous insufficiency, arterial insufficiency, and pressure over time, can lead to the reduced reparation capacity of skin injuries, which can lead to non-healing ulcers.

A non-healing ulcer, however, should not be regarded as a disease, but rather as a symptom of an underlying state. Bacteria will colonize within the ulcer if the protective barrier of the skin is broken. Therefore, the appearance of a chronic ulcer depends on several factors (Table 1). These factors also contribute to the development of infections in the ulcer.^16^ Bacteria are always found in chronic ulcers. There are often multiple types of bacteria observed within a single ulcer.

**Table 1:** Factors affecting appearance of a chronic ulcer

**Systemic factors**	**Local factors**
Metabolic diseases, such as diabetes mellitus	Size of the ulcer
Systemic diseases, such as rheumatic diseases	Age of the ulcer
Other forms of chronic disease, such as HIV infection	Location of the ulcer
Old age	Local circulation
Malnutrition / poor diet	Necrosis
Alcohol / narcotics abuse	Suppuration and maceration
Medicines, such as steroids, oestrogens, and vitamin K antagonists	Edema
Smoking	Exposed bones or capsules

For example, the flora usually found in cases of venous ulcers of the legs include *Staphylococcus aureus *(90.5%), *Enterococcus faecalis *(71.7%), and *Pseudomonas aeruginosa *(52.2%).^17^

The bacterial flora found in a non-healing ulcer change as the ulcer ages. Staphylococci and Streptococci bacteria are normally found in new ulcers, while gram negative mixed flora are often found in older ulcers. In addition, different types of ulcers are influenced by different types of bacteria. For example, a clinical infection will develop in 60% of diabetic foot ulcers but only 20% of venous leg ulcers that are colonized by *Staphylococcus aureus*.^18^ Between 1.6 and 4.4 species of bacteria are found per ulcer by conventional culturing methods.^7^ However, molecular biological methods suggest that even more species of bacteria are present in the average ulcer.^8^

The number of ulcers with anaerobic bacterial growth is estimated to be between 25% and 82%. The most common anaerobic bacterial species are *Peptostreptococcus *and *Prevotalla*.^19^^-^^21^ Recent research has indicated that the presence of bacterial biofilm contributes to the development of chronic ulcers. Studies performed earlier have shown that biofilm is present in 60% of chronic ulcers but only 6% of acute ulcers.^22^ This supports the view that biofilm probably plays an important role in the formation of chronic ulcerations.^22^

*Treatment and prevention*

*Wound Treatment:* Hyperbaric Oxygen Therapy is used to treat very serious wounds. The patient breathes 100% oxygen in a pressurized chamber for 90-120 minutes. The oxygen dissolves into the blood and is distributed throughout the body, providing extra oxygen to the cells attempting to heal the wound. Hyperbaric oxygen treatments have been found to increase the rate of collagen deposition, angiogenesis, and bacterial clearance. Another benefit is that, if the wound environment has more oxygen, certain types of bacteria that cause serious infections cannot grow. This method has been used for many years in treating difficult or complicated, non-healing wounds.^23^

It is well recognized as a very effective treatment. Skin ulcers may take a very long time to heal. Treatment is typically to avoid the ulcer getting infected, remove any excess discharge, maintain a moist wound environment, control the edema, and ease pain caused by nerve and tissue damage. Topical antibiotics are normally used to prevent the ulcer getting infected, and the wound or ulcer is usually kept clear of dead tissue through surgical debridement Whirlpool Therapy is used by physical therapists once or twice daily for about 20 minutes during the inflammatory stage of healing to enhance circulation and bring more oxygen into the wound area. The whirlpool also softens and loosens dead tissue and cleanses the wound.^24^

Ultrasound treatment uses mechanical vibration delivered at a frequency above the range of human hearing. Physical therapists report that covering the wound area with a hydrogel film and applying ultrasound during the inflammatory and proliferative stages stimulates the cells involved in wound healing and also warms the tissue, enhancing healing by improving circulation. Electrical Stimulation mimics the body’s own bioelectric system that influences wound healing by attracting repair cells, changing the permeability of cell membranes, and therefore affecting secretions and orienting cell structures.^25^

*Nutritional supplements*

Research has shown that certain nutrients such as *Aloe vera* and vitamin C play key roles in wound healing. The typical Western diet is deficient in these nutrients.^26^^,^^27^ Injury significantly increases the need for the amino acid arginine, which is essential for a variety of metabolic functions arginine stimulates the cell-mediated immune response and protects against bacterial challenges.^28^ The amino acid glutamine is an important substrate for rapidly proliferating cells, including lymphocytes (white blood cells). It is also the major amino acid lost during muscle protein catabolism in the initial response to injury.^28^ *Aloe vera* provides the micronutrients required for protein synthesis. Its many components work together to reduce inflammation and pain, promote healing, and stop infection. Aloe can be applied topically to wounds and taken internally for both skin wounds and gastrointestinal ulcers.^26^

Curcumin is an extract of the spice turmeric, known to have antioxidant properties and other health benefits. In Indian medicine, curcumin is used to reduce inflammation and treat wounds and skin ulcers. Topical application of curcumin encourages wound remodeling via effects on transforming growth factor-beta (TGF-b). It also improves reepitheliazation (new skin formation) and migration of cells such as myofibroblasts, fibroblasts, and macrophages, necessary for healing at the wound site.^29^^,^^30^

Bromelain is found in pineapple and contains a proteolytic enzyme with the ability to break down or dissolve proteins. This mechanism of action can be helpful in chronic wounds or wounds having too much scar tissue. According to the PDR for Nutritional Supplements, bromelain speeds up healing time after surgical procedures.^31^ A German physician first observed the role of copper in healing too.^32^ The immune system is adversely affected by even moderate degrees of zinc deficiency. Severe zinc deficiency depresses immune function. Zinc is required for the development and activation of T-lymphocytes, a kind of white blood cell that helps fight infection. Zinc can be used topically or orally to encourage wound healing and plays a well-documented role in wound healing.^33^

Vitamin C is crucial for the proper function of the enzyme protocollagen hydroxylase which produces collagen, the primary constituent of the granulation tissue that heals a wound and the key component in blood vessel walls. A published review stated that vitamin C plays a variety of roles in the prevention and treatment of cancer, including stimulating the immune system and enhancing wound healing. Wound healing requires more vitamin C than diet alone can easily provide. It must be replenished daily because it is water-soluble; any excess is excreted rather than stored.^34^ Vitamin B5 (Pantothenic Acid) improves healing by encouraging the migration of cells into the wounded area.^35^ Vitamin A is important for tissue synthesis and enhances resistance to infection.^36^ Vitamin B-Complex are needed for cell proliferation and for the replacement and maturation of red blood cells lost through bleeding.^37^

*Different types of discharges from ulcer are*

Serous, usually is seen in healing ulcers, while purulent is seen in infected ulcer. Yellow creamy discharge is observed in Staphylococcal infection; bloody opalescent discharge in Streptococcal infection, while greenish discharge is seen in Pseudomonas ulcers.^4^

*Ulcer grading: Wagner’s grading of ulcer follow*

Grade Description: 0: Pre-ulcerative lesion or healed ulcer, 1: Superficial ulcer, 2: Ulcer deeper to subcutaneous tissue exposing soft tissue or ulcer bone, 3: Abscess Formation Underneath, osteomyelitis, 4: Gangrene of part of tissues, limb or foot, and 5: Gangrene of entire one area or foot. Bloody (sanguineous) is usually seen in malignantulcers and in healing ulcers with healthy granulation tissue, Seropurulent, Serosanguinous, Serous with sulphur granules, seen in actinomycosis, Yellowish, as seen in tuberculous ulcer.^4^

*Complications*

**Fig. 2:**
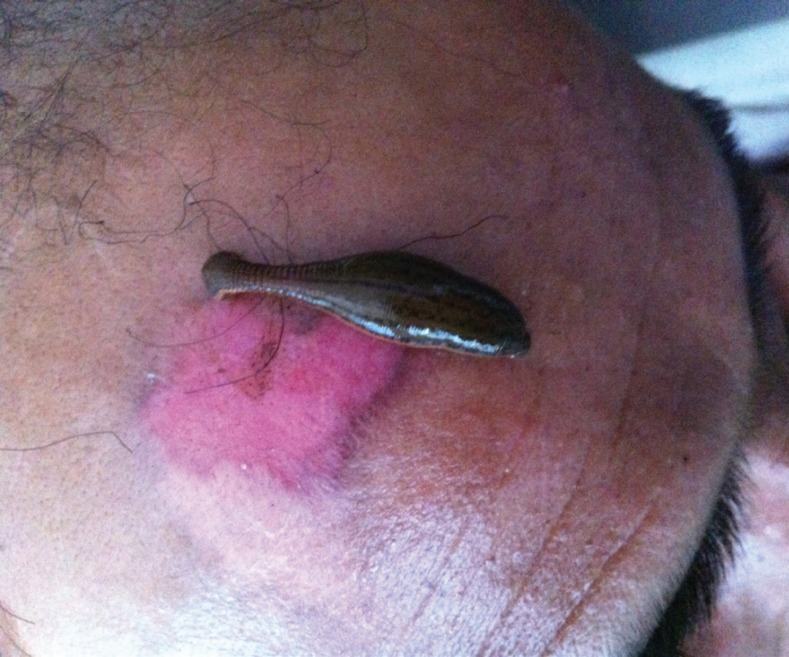
Application of leech on chronic non-healing ulcer.

**Fig. 3:**
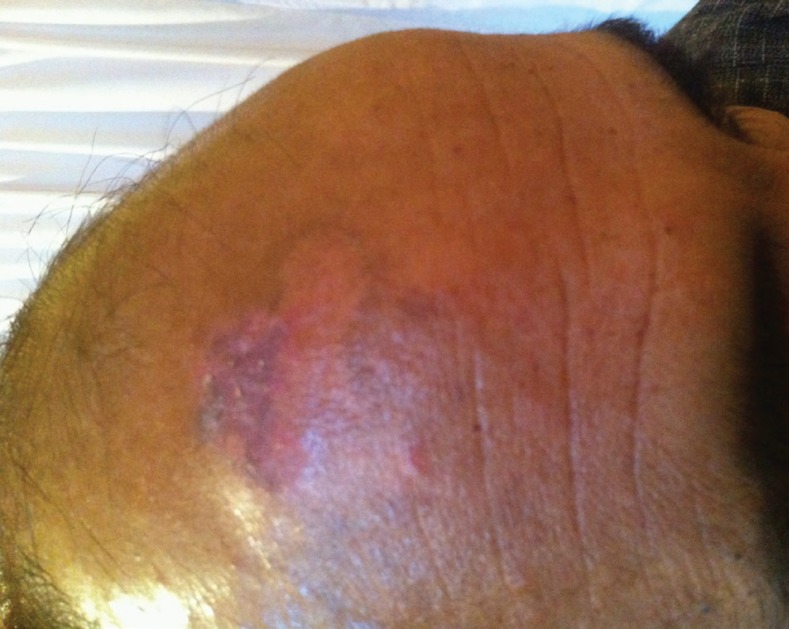
Partially healed chronic ulcer

**Fig. 4:**
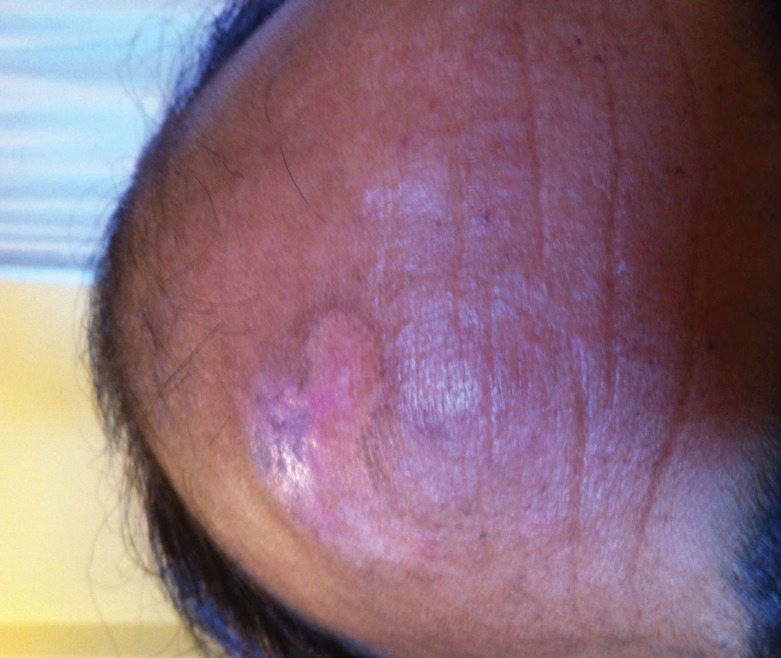
Completely healed chronic non-healing ulcer after heridotherapy

With any type of wound– even seemingly minor injuries– there is always danger of rapid multiplication of bacteria. The elderly and persons with reduced immunity are at great risk for wound-related infections. Once bacteria escape from the primary location of a wound, they enter the blood. This condition is commonly called blood poisoning, septicemia, sepsis, or septic shock. Sepsis is always a serious, life-threatening condition, with 56% mortality. In the United States, sepsis occurs annually in some three cases per 1,000 population. In sepsis and septic shock, circulation is reduced; blood pressure is markedly reduced, causing vital organs to receive reduced blood supply; heart, kidney, and liver functions are reduced or show signs of shutdown (multiple organ failure); and abnormal bleeding can develop. Symptoms of septicemia and septic shock are sudden onset of illness, high fever, chills, rapid breathing, headache, and altered mental state.^38^^,^^39^ Zaidi (2016) reported an option in treatment of poorly healing wounds with hirudotherapy (Figure 2-4).^40^

## DISCUSSION

Clinicians’ understanding of and ability to achieve wound healing has increased significantly over the past few years, particularly as a result of advances in molecular biology such as the use of growth factors, the ability to grow cells in vitro and the development of bioengineered tissue.^41^^-^^43^ Knowledge of scarring has also increased fundamentally.^44^^-^^47^ some promising results have been obtained using epidermal growth factor.^48^ and keratinocyte growth factor-2^49^ for venous ulcers, and fibroblast growth factor^50^ and platelet-derived growth factor (PDGF) for pressure ulcers.^51^^,^^52^

It is also possible that closer attention should have been paid to appropriately preparing the chronic wound before treatment with the growth factor being tested. Notably, there is evidence that the aggressive approach to surgical debridement taken in the initial PDGF trial for diabetic neuropathic ulcers seems to have worked synergistically with the application of the growth factor. bioengineered skin products or skin equivalents have become available for the treatment of acute and chronic wounds as well as burns. Since the initial use of keratinocyte sheets,^21^^,^^22^^,^^41^ several more complex constructs have been developed and tested in human wounds. Skin equivalents may contain living cells, such as fibroblasts or keratinocytes, or both,^2^^,^^41^^-^^43^ while others are made of acellular materials or extracts of living cells.^53^

Saline-soaked gauze and off-loading have been accepted by the Food and Drug Administration as the control. Bioengineered skin may work by delivering living cells which are known as a ‘smart material’ because they are capable of adapting to their environment. There is evidence that some of these living constructs are able to release growth factors and cytokines.^54^^,^^55^ The technology to introduce certain genes into wounds by a variety of physical means or biological vectors, including viruses, has existed for some time. Work with gene therapy in relation to wounds has been done in experimental animal models.^56^

There are promising indications that certain approaches may work in humans. For example, the introduction of naked plasmid DNA encoding the gene for vascular endothelial growth factor Pluripotential stem cells (PSCs), the precursors to all more specialized stem cells, are capable of differentiating into a variety of cell types, including fibroblasts, endothelial cells and keratinocytes, all of which are critical cellular components for healing. Although most PSCs are derived from human embryonic research, which is the subject of some controversy, pluripotential mesenchymal stem cells, which are the source of new connective tissue, may be present in bone marrow.^57^ (VEGF) has been reported to enhance healing and angiogenesis in selected patients with ulcers resulting from arterial insufficiency.^56^
